# A highly emissive AIE-active luminophore exhibiting deep-red to near-infrared piezochromism and high-quality lasing†

**DOI:** 10.1039/d0sc01095b

**Published:** 2020-04-03

**Authors:** Chunyan Lv, Wangwang Liu, Qing Luo, Haiyan Yi, Huakang Yu, Zhongmin Yang, Bo Zou, Yujian Zhang

**Affiliations:** Department of Materials Chemistry, Huzhou University East 2nd Ring Rd. No. 759 Huzhou 313000 People's Republic of China sciencezyj@foxmail.com; South China University of Technology Guangzhou 510640 People's Republic of China hkyu@scut.edu.cn; State Key Laboratory of Superhard Materials, College of Physics, Jilin University Qianjin Street 2699 Changchun 130012 People's Republic of China zoubo@jlu.edu.cn

## Abstract

Further development of high-efficiency and low-cost organic fluorescent materials is intrinsically hampered by the energy gap law and spin statistics, especially in the near-infrared (NIR) region. Here we design a novel building block with aggregation-induced emission (AIE) activity for realizing highly efficient luminophores covering the deep-red and NIR region, which originates from an increase in the orbital overlap and electron-withdrawing ability. An organic donor–acceptor molecule (**BPMT**) with the building block is prepared and can readily form J-type molecular columns with multiple C–H⋯N/O interactions. Notably, such synthesized materials can emit fluorescence centered at 701 nm with extremely high photoluminescence quantum yields (PLQYs) of 48.7%. Experimental and theoretical investigations reveal that the formation of the hybridized local and charge-transfer (HLCT) state and substantial C–H⋯N/O interactions contribute to a fast radiative decay rate and a slow nonradiative decay rate, respectively, resulting in high PLQYs in the solid state covering the NIR range. Remarkably, such **BPMT** crystals, as a first example, reveal strong-penetrability piezochromism along with a distinct PL change from the deep-red (*λ*_max_ = 704 nm) to NIR (*λ*_max_ = 821 nm) region. Moreover, such typical AIE-active luminophores are demonstrated to be a good candidate as a lasing medium. Together with epoxy resin by a self-assembly method, a microlaser is successfully illustrated with a lasing wavelength of 735.2 nm at a threshold of 22.3 kW cm^−2^. These results provide a promising approach to extend the contents of deep-red/NIR luminophores and open a new avenue to enable applications ranging from chemical sensing to lasing.

## Abstract 2

A HLCT-type luminophore is prepared with bright deep-red fluorescence, showing high-performance piezochromism and lasing.

## Graphical abstract

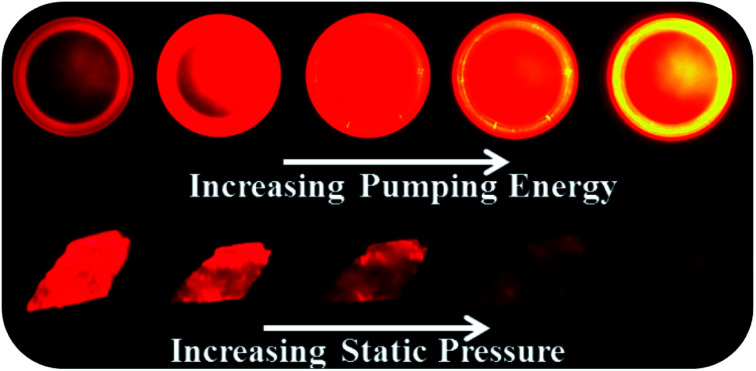

## 1. Introduction

Near-infrared (NIR) light-emitting materials have attracted growing attention in recent years, with diverse applications including biotherapy, night-vision, up-conversion lasers and so on.^1^ Compared with inorganic NIR materials, organic NIR materials are inexpensive and feasible for fabrication, and their diverse structures enable fruitful possibilities for the abovementioned applications. In recent years, increasing efforts have been devoted to developing highly efficient pure organic NIR materials. Although metal complex phosphorescence materials have already been proven to be an effective solution to overcome the low efficiency problem,^2^ the cost and preparation problem remain since the fabrication process includes noble metal complex material systems. Therefore, low-cost fabrication techniques for pure organic materials without any noble metals are still desirable.

In general, one promising method to construct NIR luminophores is based on the donor–acceptor (D–A) molecular architecture.^3^ However, most D–A molecular systems exhibit a very low photoluminescence efficiency (PL). This point is easy to understand. First, the charge-transfer (CT) emissive state usually has a low (even zero) radiative rate as a result of the significant spatial separation between the highest occupied molecular orbital (HOMO) and lowest unoccupied molecular orbital (LUMO) in the D–A system. Second, the relatively low PL efficiency usually comes from an intrinsic limitation of the “energy gap law”.^4^ Therefore, the nonradiative rate of a luminophore will increase exponentially as the energy gap is reduced to the NIR region due to the increased overlap between the excited and ground state vibrational manifolds. In principle, an increase in molecular rigidity would contribute to the enhanced radiative rate,^1*c*^ although this strategy generally corresponds to the increased molecular planarity, ultimately favouring H-type aggregation quenching. To tackle these problems, two effective methods have been proposed. One method is *via* aggregation-induced emission (AIE) raised by B. Tang.^5^ This method sterically limits the π–π molecular stacking responsible for aggregation quenching and also restricts the intramolecular motions of peripheral rings. Another method is to directly regulate the excited state component for an enhanced radiative transition rate of a D–A luminophore, known as the hybridized local and charge-transfer (HLCT) method.^6^ For HLCT-active luminophores, the locally excited (LE) state component usually possesses a large transition dipole moment and a large orbit overlap between hole and electron moieties, which thereby effectively enhance the PL quantum yields (PLQYs). Recently, the HLCT method has been applied to the molecular design for highly efficient and sensitive chemical sensing.^7^ It is natural to adopt the principle of the HLCT state for designing NIR luminophores, where the LE state could lead to high PLQYs and the charge transfer (CT) state could contribute to the red-shifted emission.

**Scheme 1:**
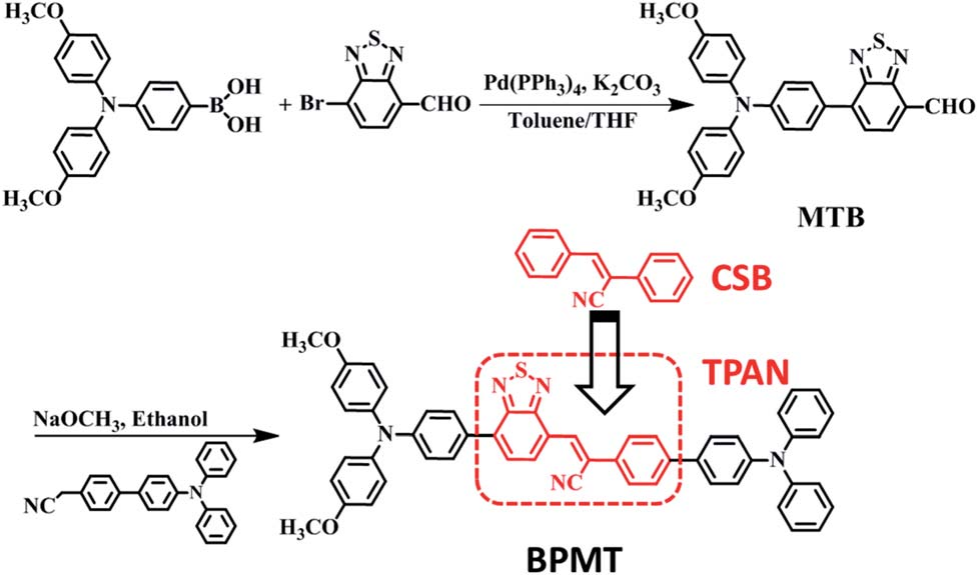
The synthetic route of **BPMT**.

α-Cyanostilbene (**CSB**, Scheme 1), as an AIE-active building block, was developed by Park *et al.* in 2002 with the advantages of facile preparation, easy modification, and outstanding optical features.^8^ As the cyano group has an electron-withdrawing character, triphenylamine (TPA)-modified **CSB** derivatives usually exhibited a typical CT behaviour. However, their fluorescence rarely covered the deep-red/NIR region.^9^ Moreover, wavefunction overlap between “hole” and “particle” is clearly observed on the **CSB** unit, facilitating the construction of the HLCT state.^7*b*^ In this study, for developing deep-red/NIR PL materials, we constructed a new-style building block, **TPAN**, as shown in Scheme 1, by the introduction of a benzo[*c*][1,2,5]thiadiazole (**BTA**) unit. This strategy is based on the following considerations. First, one could expect that a larger planar **BTA** group would contribute to an increase in orbital overlap, which is beneficial to enhance radiative transition and PLQYs. Second, **TPAN** tends to present a stronger electron-withdrawing ability, which can lead to a stronger CT and a red-shifted PL peak. After systematic spectral experiments and theoretical analyses, it is found that TPA-modified **TPAN** derivatives (**BPMTs**) show unique HLCT features and a typical AIE behaviour. In the crystalline state, the propeller-shaped TPA unit sterically restricts the π–π molecular stacking of **BPMT**, thereby suppressing the aggregation-caused quenching significantly. Interestingly, the rod-like crystals emit bright fluorescence covering the deep-red and NIR region with an extremely high PLQY of 48.7%. Moreover, the crystal shows strong-penetrability piezochromism under high pressure. To the best of our knowledge, this is a first example of a fluorophore showing piezochromic behaviour covering the deep-red and NIR region (>700 nm).^1*f*,10^ Inspired by its excellent AIE behaviour, a **BPMT**-based laser was successfully achieved.

## 2. Results and discussion

### 2.1 Molecular design and synthesis

**Fig. 1:**
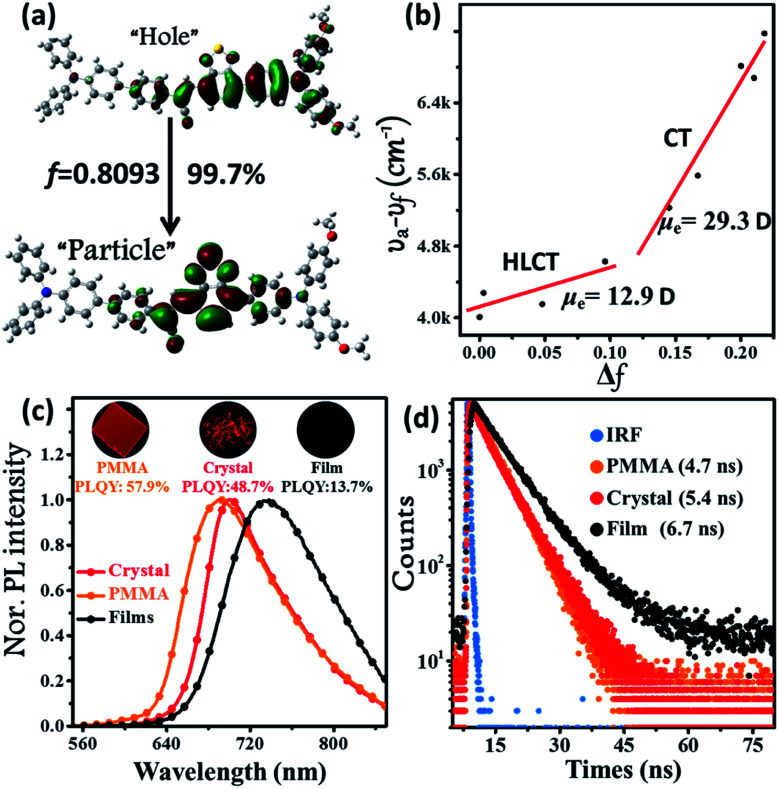
(a) Natural transition orbitals (NTOs, S_0_ → S_1_) of the **BPMT** single crystal structure using the TD/M06-2X/6-31g(d,p) method, where *f* is the oscillator strength. (b) Linear correlation of the orientation polarization (Δ*f*) of solvent media with the Stokes shift (*ν*_a_ − *ν*_f_; a: absorption; *f*: fluorescence) for the **BPMT** (see Table S1† for data; the lines in low- and high-polarity regions represent the HLCT and CT states, respectively). (c) Steady-state PL spectra and (d) time-resolved PL decay curves of **BPMT** in various states (1 %wt/wt dye-doped film, crystalline powder, and spin-coated film). The inset images in Fig. 2c show the corresponding PL photographs under 365 nm UV light.

The synthetic route of the NIR chromophore **BPMT** is illustrated in Scheme 1, and more detailed procedures are shown in the Experimental section. Intermediate **MTB** was obtained through a Suzuki coupling reaction of 7-bromobenzo[*c*][1,2,5]thiadiazole-4-carbaldehyde and (4-(bis(4-methoxyphenyl)amino)phenyl)boronic acid. Then, **BPMT** was prepared by the Claisen–Schmidt condensation reaction of **MTB** with TPA-modified phenylacetonitrile. Lastly, all the structures were spectroscopically characterized using nuclear magnetic resonance spectroscopy (NMR) and mass spectrometry (MS), together with X-ray crystallography. And all the measurement results are in good agreement with their expected structures. To investigate the PL process of **TPAN**-based derivatives at the molecular level, the ground-state and excited-state properties were carefully studied at the TD-M062X/6-31g(d,p) level. The optimized geometries of the **TPAN** unit in the gas phase adopted a planar conformation with very small twisted angles (less than 28°), which is consistent with its crystal state (see below). This feature enables large π-conjugation, leading to PL red shift and PLQY enhancement in the **BPMT** chromophore. More importantly, compared with that of **CSB**-based derivatives,^7*b*,11^ hole–electron overlap of **BPMT** on the acceptor unit (**TPAN**) is significantly increased due to the introduction of **BTA** (Fig. 1a). The NTOs of the S_1_ excited state demonstrate a typical HLCT character^6,12^ and are also prone to the formation of high oscillator strength (*f* = 0.8903). Introduction of the CT behavior from the triphenylamine (TPA) to **TPAN** leads to an additional red-shifted PL peak. Moreover, multiple inter-molecular noncovalent interactions (C–H⋯O, C–H⋯N, *etc.*) can be constructed in the aggregated state through the introduction of methoxyl and cyanoyl groups,^13^ which contribute to the restriction of intramolecular motions (RIMs) and enhancement of PLQYs.

### 2.2 Solvatochromic effects

**Fig. 2:**
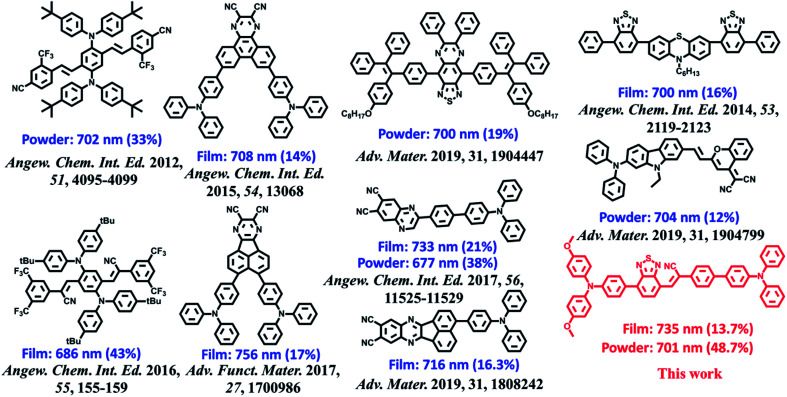
Molecular structures of **BPMT** and the state-of-the-art NIR emitting materials and their corresponding PL peaks and PLQYs of the crystalline powder and amorphous films.

The ultraviolet-visible (UV-vis) absorption and PL spectra of the luminophore **BPMT** in different polar solvents are depicted in Fig. S1 and S2,† and the corresponding data are summarized in Table S2.† The UV-vis spectra of **BPMT** involved two characteristic absorption peaks (Fig. S1†). The low-energy peak (approximately 520 nm) could be ascribed to the S_0_ → S_1_ transition (CT-like character). And the absorption spectra of the **BPMT** dye in different solvents did not show obvious changes. For the PL spectra in different solutions, considerable red-shift was observed from 660 nm (hexane) to 817 nm (dichloromethane, DCM), accompanied by drastically decreased PLQYs from 83% (hexane) to <1% (DMF), as shown in Fig. S2.† This red-shift revealed that the low-lying excited state, S_1_, of **BPMT** had a certain CT-like character.^3,6^ The properties of the excited state were further investigated from the slope of the Stokes shifts (*ν*_a_ − *ν*_f_) *versus* the polarity of the solvents (Δ*f*) according to the Lippert-Mataga model. As shown in Fig. 1b, a clear two-section line was found, revealing the existence of two different excited-state characteristics. The excited-state dipole moment *μ*_e_ of **BPMT** was evaluated to be 29.3 D in high-polarity solvents, arising mainly from a CT-like state. However, in low-polarity solvents, *μ*_e_ became as small as 12.9 D, which is slightly higher than that of the typical LE states. This finding corresponds to the relatively high PLQYs of **BPMT** in low-polarity solvents (see Table S2†). These results, in combination with the clear red-shift of the PL spectra, demonstrated that the S_1_ state of **BPMT** was an LE-like state involving a slight CT component. Moreover, the time-resolved decayed lifetime curves of **BPMT** revealed single-exponential lifetimes of 4.6 and 3.0 ns in hexane and butyl ether, respectively (Fig. S3†). These facts indicated that the S_1_ state of **BPMT** was hybridization of the LE and CT states (HLCT state), as confirmed in the D–A luminophores previously.^6,12^ Therefore, an LE-dominated HLCT state was formed in low-polarity solvents, which favoured the highly bright fluorescence of **BPMT** extended to the NIR region. In principle, the “environment” of poly(methyl methacrylate) (PMMA) was similar to that of luminophore in low-polarity solvents. As depicted in Fig. 1c, the doped PMMA film emitted deep-red fluorescence (692 nm) with a high PLQY of 57.9%. It can be inferred that the HLCT state exists in such low-polarity solid phases. Notably, the crystalline powders of **BPMT** revealed excellent PL performances with significantly red-shifted fluorescence covering the deep-red and NIR region and an extremely high PLQY of 48.7%, which is a stand-out result among the NIR emissive luminophores (Fig. 2). As depicted in Table S2,† the Δ*f* of the **BPMT** crystals (4689 cm^−1^) was close to that in butyl ether (4628 cm^−1^), which implied that the HLCT behaviour occurred in the crystalline powders. Moreover, a single-exponential PL decay process of crystals was observed with a lifetime of 5.4 ns (Fig. 1d). Calculated radiative rates (*k*_r_ = PLQYs/*τ*) were as large as 9 × 10^7^ s^−1^, consistent with the large oscillator strength (Fig. 1a). From these facts, one can conclude that the HLCT state here opens the radiative channel and brings in high PLQYs of the crystalline powders.

### 2.3 Aggregation-enhanced emission

**Fig. 3:**
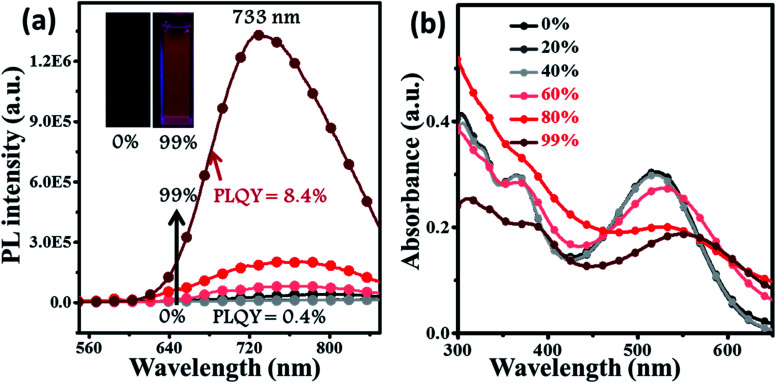
The PL (a) and absorption (b) spectra of **BPMT** (10 μm) in THF/water mixtures with different water fractions (*f*_w_). The inset in (a): PL photographs (*f*_w_ = 0 and 99%) under a UV lamp.

**Fig. 4:**
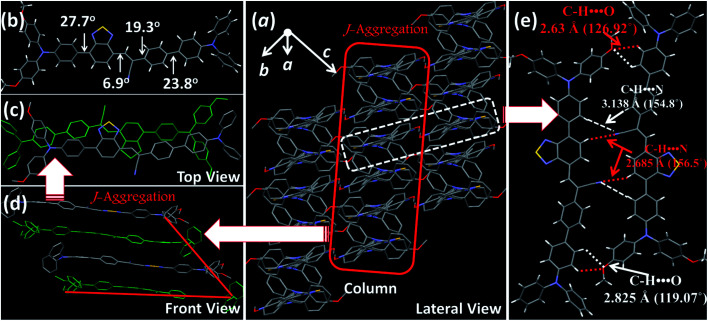
Crystal structures of **BPMT**: (a) lateral view of the column arrangement; (b) the dihedral angles of the **BPMT** molecule in the crystalline state; (c) front view of the anti-parallel arrangement with *J*-aggregation; (d) top view of the *J*-aggregated column; and (e) the illustration of C–H⋯N/O hydrogen bond interactions.

For deep-red/NIR fluorescence, one should also consider the nonradiative processes of the excited state except for the above radiative routes. It is noted that the luminophore **BPMT** was almost non-radiative in high-polarity solvents, such as tetrahydrofuran (THF), acetonitrile, and acetone, due to its CT character. However, the PLQYs of its film was considerably high (13.7%), in accordance with its AIE feature. Indeed, for a typical AIE process, when water was continuously added into the THF solution of **BPMT**, its PL intensity was clearly enhanced (Fig. 3a), with a corresponding increase in the PLQYs from 0.4% to 8.4%. According to previous reports, such AIE behaviour of **BPMT** reflected not only a change in the excited state^1*f*,14^ but also a typical restriction of the nonradiative channel. As illustrated in Fig. 3b, in solutions with larger water volume fractions, the absorption spectra exhibited an obvious red-shift, showing that *J*-aggregation might occur in the aggregates of **BPMT**.^8^ To further investigate the photophysical properties of **BPMT**, especially the supramolecular interactions for the AIE behaviour, a rod-like single crystal was obtained by the slow evaporation of the saturated CH_2_Cl_2_ solution at room temperature. As shown in Fig. 4b, the **TPAN** unit was almost planar with a dihedral torsional angle of less than 20°, consistent with the oscillator strength (*f* = 0.8093, Fig. 1a). As a result, the planar conformation allowed a large π-conjugation with the coexistence of LE and CT states, which leads to red-shifted monomolecular fluorescence with high PLQYs. Notably, the molecules were packed into J-type molecular columns along the axis, which contributed to a further red-shift of the PL spectra, even to the NIR region. It needs to be pointed out here that the large radiative transition of unimolecules was not affected by aggregation factors.^15^ Moreover, there were multiple C–H⋯N/O interactions between adjacent molecules along the *b* axis, as shown in Fig. 4e, which fixed the ‘‘molecular columns’’ inside final crystal structures (Fig. 4a). And we should mention that this large number of multiple hydrogen bond interactions restricted the intramolecular rotation (the nonradiative decay channel) of the **BPMT** luminophore, which activated its strong PL intensity in aggregated states. Therefore, the HLCT behaviour, in combination with the restriction of the nonradiative channel, was responsible for the high PLQYs of the crystalline powder.

### 2.4 Piezochromic properties

**Fig. 5:**
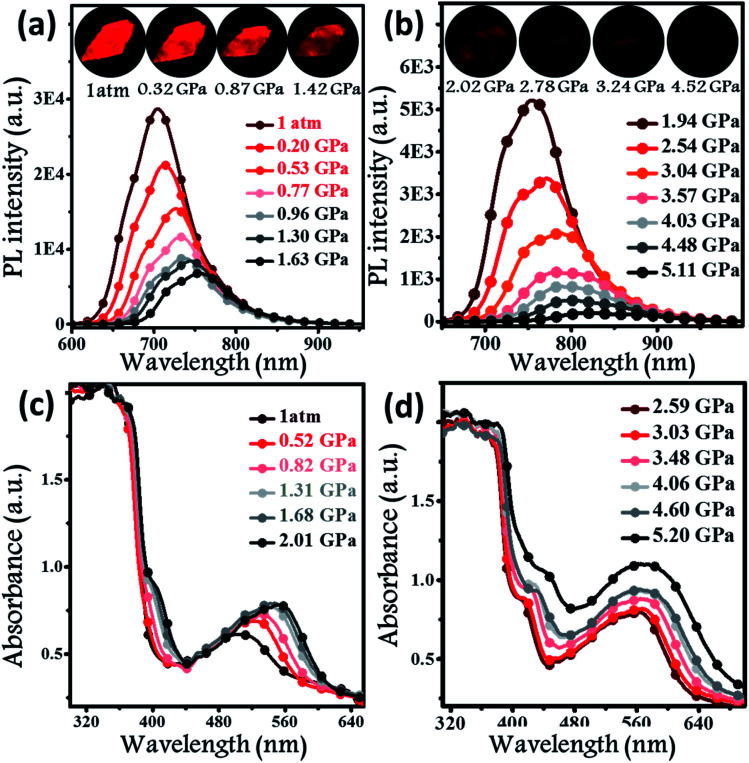
*In situ* PL spectra (a and b) and absorption spectra (c and d) of **BPMT** crystals under different hydrostatic pressures. Inset illustrates the PL photographs of a small piece of the **BPMT** crystal at various pressures (from 1 atm to 4.52 GPa).

Hydrostatic pressure, as a common natural stimulus, can alter the PL intensity or/and colour through a phenomenon referred to as piezochromism.^16^ Presently, the practical requirements for deformation detection, haptic sensing, and flaw detection have promoted research into piezochromic materials (PCMs) with strong penetrability and clear PL spectral shifts. Longer wavelengths, *e.g.*, the NIR fluorescence, indicates the possibility of a stronger penetrability.^1*c*,3*a*,17^ There are rare examples of PCMs with PL spectra longer than 700 nm, even extended to the NIR region. Regarding the excellent PL properties, the potential application of a **BPMT** luminophore was further investigated under high pressure. In Fig. 5a and b, brightness of fluorescence photographs for a small piece of a **BPMT** crystal gradually decreased. In addition, fluorescence peaks shifted from approximately 704 to 821 nm in a range of pressures from 1 atm to 5.1 GPa. Upon decompression, the corresponding PL spectra returned to their original state (Fig. S5†). Clearly, **BPMT** revealed reversible piezochromic luminescence under high pressure. This is to further find out that the position of the PL peaks displayed a linear relationship with respect to hydrostatic pressure (Fig. S6†). The piezochromic sensitivity, *η*, was estimated to be as high as 20.8 nm GPa^−1^. These results demonstrate that the crystal **BPMT** is a good candidate as a colorimetric sensor with strong penetrability and high sensitivity. As depicted in Fig. 5c and d, upon increasing the pressure, the absorption sidebands gradually redshifted to longer wavelengths by 114 nm. The corresponding energy gap decreased from 2.19 eV (566 nm) to 1.82 eV (680 nm). This implied that the red-shifted spectra of **BPMT** crystals were ascribed to conformational planarization.^1*f*,18^ As depicted in Fig. S7,† the excitation energies of molecular configurations with decreasing dihedral angles (*η*) were calculated. The rotation of the TPA unit to reduce the dihedral angle resulted in a decrease in the S_1_ excitation energy, which corresponds to the PL redshift during compression. This result further confirmed that the planarity of molecular conformation led to the red-shifted emission. Moreover, the larger orbital overlap between “hole” and “particle” barely altered, which demonstrated that the HLCT state still remained under high pressure. The S_1_ oscillator strength of the conformation with ∼31.7° dihedral angles (0.7668) was smaller than that with 22.7° dihedral angles (0.8400, see Fig. S7†), showing that the radiative transition rate of the CT state is higher in a more planar conformation, contributing to the slowing of PL quenching under high pressure.

IR spectra in the range of 800–3150 cm^−1^ at high pressure were investigated to track the alteration of the molecular structure. As shown in Fig. S8a,† absorption peaks at 3039 and 2211 cm^−1^ could be readily attributed to the stretching vibrations of aromatic C–H and C≡N units, respectively. The stretching vibration of –C–O– was located at 1239 cm^−1^. As mentioned above, two kinds of supramolecular interactions are presented, C–H⋯O effects and C–H⋯N hydrogen bonds, causing the J-type aggregation of **BPMT** molecules, which has a relatively low nonradioactive rate. Fig. S8b† reveals that their IR peaks from the stretching vibration show a clear blue-shift during compression, implying a decrease in the interatomic distances of the C–H⋯N/O hydrogen bond. Namely, the *J*-aggregated column was more closely packed when the hydrostatic pressure was applied. This change enhancing the intermolecular interactions, in combination with the conformational planarization, resulted in a red-shift of the PL wavelength and a decrease in PL intensity.

### 2.5 Whispering gallery mode lasing

**Fig. 6:**
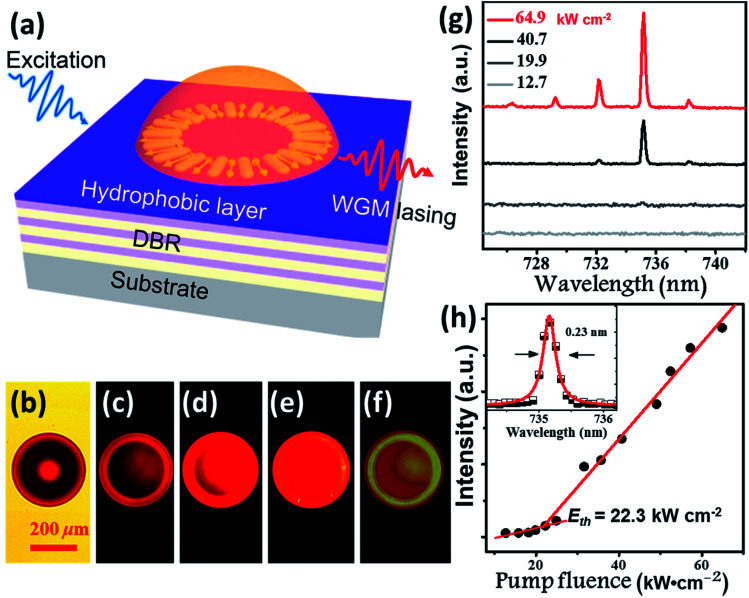
Whispering gallery mode lasing from the **BPMT**-doped hemispherical microresonators. (a) Schematic of the optically pumped hemispherical microresonators on a DBR substrate. (b) Bright-field and (c–f) fluorescence images of the hemisphere with a 36 μm diameter with increasing pump intensities. The scale bar is 200 μm. (g) Collected spectra with pump intensities at 12.7, 19.9, 40.7, and 64.9 kW cm^−2^. (h) Integrated output intensity as a function of the pump intensity; the inset shows Lorentz fitting (red line) of a measured lasing spectral peak mode at 735.2 nm.

Benefitting from their excellent AIE properties, we successfully realized the lasing of **BPMT** materials. **BPMT** was dissolved in DCM together with epoxy resin (Araldite 506, Sigma-Aldrich). The PL spectra of the prepared mixture show an emission peak at 695 nm with a full width at half maximum (FWHM) of 110 nm, as shown in Fig. S9.† As a result of the hydrophobic effect and surface tension force, **BPMT**-doped hemispherical microresonators were fabricated *via* a self-assembly process on a distributed Bragg reflector (DBR) mirror surface by following previous literature (see the Experimental section for more details).^19^ To achieve lasing, the fabricated hemispherical microresonators were excited under a 10× objective (NA = 0.3) of a home-made micro-PL (μ-PL) system by a nanosecond laser (351 nm, 10 ns duration, 100 Hz), as schematically shown in Fig. 6a. Fig. 6b shows a bright-field optical microscopy image of a typical hemispherical microresonator, and Fig. 6c–f presents fluorescence microscope images with increasing pump intensities. For pump power above the lasing threshold, one could easily find a shining outer boundary of such a hemispherical microresonator, revealing the nature of the whispering gallery mode (WGM) here. Fig. 6g shows a typical measured power-dependent spectra of the **BMPT**-doped hemispherical microresonator with a 36 μm diameter. Along with increased pump power, multiple lasing peaks at approximately 730 nm become clear above the lasing threshold. The corresponding power-dependent plots of the integrated intensity of the lasing peaks as a function of the excitation power are shown in Fig. 6h. Fig. 6h presents a clear superlinear curve, which indicates the transition from PL emission to lasing at a pump threshold of 22.3 kW cm^−2^. The quality factor is estimated to be 3200 for a lasing wavelength of 735.2 nm with a linewidth of 0.23 nm (see the inset in Fig. 6h), suggesting a high-quality microlaser. Like the strain-induced laser modulation,^20^ one may readily expect that the lasing behavior could be efficiently modulated due to piezochromism effects. This could be very interesting and should be carefully investigated in future work.

## 3. Conclusion

In summary, a novel AIE-active building block (**TPAN**) as an electron-withdrawing unit was successfully developed to achieve high-brightness fluorescence covering the deep-red and NIR region. The introduction of rigid planar **BTA** into the AIE-active **TPAN** unit significantly extended the effective molecular conjugation, which could contribute to the increase in orbital overlap (LE-dominance) and red-shift of the PL spectra. As a result, the luminophore **BPMT** with such a building block, was effective at suppressing the nonradiative energy loss and emitted fluorescence covering the deep-red and NIR region with PLQYs as high as 48.7%. Endowed with distinct photophysical characteristics, relatively high PLQYs and AIE behaviour, the **BPMT** crystals were successfully used to realize strong-penetrability piezochromism (over 700 nm), almost surpassing all previously reported piezochromism performances. Finally, the **BPMT**-doped hemispheres were self-assembled on a DBR *via* a hydrophobic effect, which gave rise to the 735 nm lasing with a high-quality factor Q of 3200. This work not only puts forward a novel AIE-active building block to prepare high-efficiency NIR luminophores, but also opens up new application prospects for AIE materials.

## 4. Experimental section

### 4.1 Synthesis of **MTB**

(4-(bis(4-methoxyphenyl)amino)phenyl)boronic acid (0.77 g, 2.2 mmol), 7-bromobenzo[*c*][1,2,5]thiadiazole-4-carbaldehyde (0.49 g, 2 mmol), 20 mL of dry toluene, 30 mL of dry THF and 5 mL aqueous of K_2_CO_3_ solution (2.0 M) were placed in a 250 mL round-bottom flask. And then, Pd(PPh_3_)_4_ (104 mg, 0.09 mmol) was added. The above mixture was vigorously stirred at 100 °C for 12 hours under nitrogen. Water (25 mL) was added to quench the reaction, and the mixture was then extracted with dichloromethane. The organic solution was extracted with dichloromethane followed by purification by column chromatography on silica gel with petroleum ether/dichloromethane (2 : 1) as the eluent to offer a deep-red solid. The desired compound was obtained in 85% yield (1.4 g).

^1^H NMR (500 MHz, CDCl_3_) *δ* 10.73 (s, 1H), 8.27 (d, *J* = 7.5 Hz, 1H), 7.90 (d, *J* = 9.0 Hz, 2H), 7.82 (d, *J* = 7.5 Hz, 1H), 7.17 (d, *J* = 9.0 Hz, 4H), 7.05 (d, *J* = 9.0 Hz, 2H), 6.89 (d, *J* = 8.5 Hz, 4H), 3.82 (s, 6H); ^13^C NMR (100 MHz, CDCl_3_) 188.9, 156.6, 153.9, 153.8, 150.2, 140.1, 139.8, 134.4, 133.1, 130.4, 127.4, 127.2, 125.2, 125.1, 118.8, 114.9, 55.5; MAIDI-TOF-MS: *m*/*z*: calcd for C_27_H_21_N_3_O_3_S: 467.1298 [M]^+^, found: 467.1296.

### 4.2 Synthesis of **BPMT**

A mixture of **MTB** (1.8 g, 5 mmol) and 2-(4′-(diphenylamino)-[1,1′-biphenyl]-4-yl)acetonitrile (0.62 g, 5 mmol) in ethanol (HPLC, 40 mL) was stirred at room temperature for 5 min. And then, a small amount of NaOMe powder was added and stirred for 12 hours at room temperature. The resulting dye (**BPMT**) was filtered and repeatedly washed with EtOH solution (∼50°) to give red powders (2.1 g, 91%). ^1^H NMR (400 MHz, CDCl_3_) *δ* 8.74 (d, *J* = 7.6 Hz, 1H), 8.59 (s, 1H), 7.88 (dd, *J*_1_ = 8.0 Hz, *J*_2_ = 4.8 Hz, 4H), 7.81 (d, *J* = 8.0 Hz, 1H), 7.70 (d, *J* = 8.4 Hz, 2H), 7.53 (d, *J* = 8.4 Hz, 2H), 7.27–7.31 (m, 4H), 7.16 (d, *J* = 8.8 Hz, 10H), 7.04–7.08 (m, 4H), 6.88 (d, *J* = 8.8 Hz, 4H), 3.82 (s, 6H); ^13^C NMR (100 MHz, CDCl_3_) *δ* 156.4, 155.1, 153.2, 147.8, 147.5, 140.2, 135.8, 134.7, 132.6, 130.0, 128.1, 127.9, 127.7, 127.3, 126.6, 124.7, 124.5, 123.5, 123.2, 119.3, 118.23, 114.8, 114.8, 55.5; MAIDI-TOF-MS: *m*/*z*: calcd for C_53_H_39_N_5_O_2_S: 809.2818 [M]^+^; found: 810.2815.

### 4.3 Fabrication of **BPMT**-doped hemispherical microresonators

**BPMT**-doped hemispherical microresonators were obtained *via* a self-assembly process (hydrophobic effect and surface tension force). A commercial broadband dielectric mirror (*i.e.*, BB1-E02, Thorlabs Inc.) was used as a DBR mirror substrate, with up to 99% reflectivity in the wavelength range of 400–750 nm. The surface of the DBR substrate was treated to become hydrophobic by spin coating a layer of triethoxy (1,1*H*,2*H*,2*H*-perfluoro-1-octyl) silane (POTS, Sigma Aldrich). Dichloromethane (DCM) was used to dissolve both **BPMT** at 10 mg mL^−1^ concentration and epoxy resin (Araldite 506, Sigma-Aldrich) at 200 mg mL^−1^ concentration. The solution for the creation of hemispheres was prepared after blending and DCM solvent evaporation. To fabricate the microresonators, a fiber taper was used to deposite a drop of the aforementioned mixture onto the DBR surface. Hemispherical microresonators were immediately formed due to the hydrophobic effect and surface tension force. The size of the hemispherical microresonator can be controlled from 200 μm to 5 μm by carefully adjusting the volume of solution transferred.

## Conflicts of interest

The authors declare no competing interests.

## Supplementary Material

SC-011-D0SC01095B-s001

SC-011-D0SC01095B-s002
