# Supplementary material for: A highly emissive AIE-active luminophore exhibiting deep-red to near-infrared piezochromism and high-quality lasing
Source: Chem Sci. 2020 Apr 3;11(15):4007–15. doi: 10.1039/d0sc01095b (PMC8152686; doi:10.1039/d0sc01095b)
Supplement: SC-011-D0SC01095B-s001 [file SC-011-D0SC01095B-s001.pdf]

Electronic Supplementary Material (ESI) for Chemical Science.
This journal is © The Royal Society of Chemistry 2020

Experimental section

***Materials*:** (4-(bis(4-methoxyphenyl)amino)phenyl)boronic acid, 7-bromobenzo-[c][1,2,5]thiadiazole-4-carbaldehyde and 2-(4'-(diphenylamino)-[1,1'-biphenyl]-4-yl)acetonitrile were purchased from Alfa Aesar Co. Ltd. Other reagents were obtained from Sigma-Aldrich or Aladdin Chemicals and used without further purification. Solvents were purified according to standard laboratory methods.

***Atmospheric pressure measurements*:** ^{1}H NMR and ^{13}C NMR of the desired luminophores were recorded on a Brüker AM400 spectrometer using tetramethyl silane (TMS, δ=0 ppm) as internal standard. The PL spectra at room temperature were obtained on a SENS-9000 (Gilden Photonics, England). The digital photographs were captured by the 550D digital cameras (Canon, Japan). Powder X-ray diffraction experiments were measured on a Philips X'Pert Pro diffractometer (Netherlands). Measurements were made in a 2θ range of 5-50° at room temperature with a step of 0.02° (2θ).The scan speed was 2 degree/min. The UV-*vis* absorption spectra were obtained on a Shimadzu UV-2600 spectrophotometer (Japan). The high-resolution MALDI-TOF-MS were measured on SolariX-70FT-MS (Brüker, Germany) using CH_2Cl_2 as solvent. Time-resolved fluorescence decay spectra of the **BPMT** were performed on an Edinburgh FLS980 fluorescence spectrometer. The picosecond laser was chose as the excitation light source with its model of EPL-510. The wavelength for excitation light was set as 510 nm. The testing technique was based on TCSPC (Time-Correlated Single Photon Counting). The PL lifetime (τ) of the BPMT were obtained by fitting the decay curve with a multi-exponential decay function of $I(t) = A_1 \bullet \exp(-t/\tau_1) + A_2 \bullet \exp(-t/\tau_2) + ... + A_i \bullet \exp(-t/\tau_i)$, where A_i and τ_i represent the amplitudes and lifetimes of the individual components for multiexponential decay profiles, respectively. The time range was set as 100 ns, and the number of counts was set as 5000. Absolute PLQYs were obtained using a Quantaurus-QY measurement system (C11347-11, Hamamatsu Photonics). All solution samples were excited at the wavelengths corresponding to their individual maximum absorption peaks from the UV-visible absorption spectra, respectively (for example, 522 nm for that in Hexane, and 515 nm for that in DCM). Likewise, the solid-state samples were excited at the wavelengths corresponding to their individual maximum peaks from the PL excitation spectra (for example 525 nm for PMMA).

High-pressure measurements: A piece of crystal was placed in the hole of a T301 steel gasket with a mixture of methanol and ethanol (V/V, 4/1) for pressure transmission medium (PTM) and ruby chip as pressure calibration. The *In-situ* PL spectra at high pressure were accomplished on an Ocean Optics QE65000 pectrometer in the reflection mode. The 355 nm line of a DPSS laser(violet diode laser) with a spot size of 20 mm and a power of 10 mw was used as the excitation source. The diamond anvil cell (DAC) containing the sample was put on a Nikon fluorescence microscope to focus the laser on the sample. PL photographs of the compressed **DFPA** crystals were taken by an imaging camera (Canon EOS 5D Mark II) equipped on the fluorescence microscope. The camera can record the photographs under the same conditions including exposure time and intensity. The *In-situ* Raman spectra at high-preussure were obtained in the standard backscattering geometry with the Acton SpectraPro 2500 spectrograph. The *In-situ* UV-vis absorption spectra were measured on Ocean Optics QE65000 spectro-photometer. The *In-situ* IR microspectroscopy of crystalline powders at high pressure was performed on a Bruker Vertex80 V FTIR with KBr as the PTM.

Lasing measurements. A micro-photoluminescence (μ-PL) system was used to excite the individual BPMT-doped hemisphere. The excitation laser was a pulsed nanosecond laser with 351-nm wavelength, 200-Hz repetition-rate, and 7-ns pulse duration. The light was focused onto the sample surface by a 10X objective (TU Plan Fluor EPI P 10X, NA=0.3). The excited laser beam diameter under the microscope was estimated to be about 100 μm. The collected spectrum was recorded by a charged coupled device (CCD) and spectrometer (MAYA 2000, resolution: 0.1 nm).

Theoretical calculations**:** The geometries of all molecules were fully optimized at the SCF level of theory using the Gaussian 09[6] suite of programs package. The ground -state geometries have been optimized by using density functional theory (DFT) at m062x/6-31g(d,p) level.

Table S1. Crystal data and structure refinement for crystals **BPMT**.

Samples	**BPMT (CCDC: 1983745)**
Formula	$C_{53}H_{39}N_5O_2S$
Mr	809.95
Temperature (K)	296
Crystal system	Triclinic
Space group	P-1
Crystal size (mm)	0.19 × 0.12 × 0.08
a (Å)	10.3043(4)
b (Å)	10.5271(5)
c (Å)	21.1147(9)
α (°)	88.634(1)
β (°)	86.866(2)
γ (°)	70.331(1)
V(Å^3)	2153.50(16)
Z	2
D_{calc} (mg/m^3)	1.249
Theta Range (°)	2.19-27.47
F (000)	848.0
h, k, l_{max}	12,13,26
N_{ref}	8759
T_{min}, T_{max}	0.693, 0.745
Independent reflections	1978
Goodness-of-fit on F^2	0.982
R_{int}	0.0497
$R_1[I>2\sigma(I)]$	0.0558
$wR_2[I>2\sigma(I)]$	0.1385
R_1(all data)	0.2180
wR_2(all data)	0.1534
S	1.026

$R_1 = \Sigma||F_o| - |F_c||/\Sigma|F_o|$, $wR_2 = [\Sigma w(F_o^2 - F_c^2)^2/\Sigma w(F_o^2)^2]^{1/2}$

The change in magnitude of the dipole moment between the ground and excited states, that is, $\Delta\mu = |\mu_e - \mu_g|$ can be estimated using the Lippert–Mataga equation

$$hc(v_a - v_f) = hc(v_a^0 - v_f^0) + \frac{2(\mu_e - \mu_g)^2}{a_0^3} f(\varepsilon, n)$$

Where a_0 is the cavity radius in which the solute resides, estimated to be 6.5 Å. μ_g is the ground-state dipole moment, estimated to be 5.2 D (ωB97X at the basis set level of 6-31G**), μ_e is the excited state dipole moment. h and c are Planck's constant and the speed of light, respectively, and $f(\varepsilon, n)$ is the orientation polarizability, defined as

$$f(\varepsilon, n) = \frac{\varepsilon - 1}{2\varepsilon + 1} - \frac{n^2 - 1}{2n^2 + 1}$$

Where ε is the static dielectric constant and n is the optical refractivity index of the solvent. Through the analysis of the fitted line in low-polarity solvents, its corresponding μ_e was calculated to be 12.9 D with the slope of 5220 according to Lippert-Mataga equation. However, in high-polarity solvents, the μ_e was increased to 29.3 D with the slope of 26932.

Table S2 Detailed photo-physical data of **BPMT** in the different solvents.

**Solvents**	ε	n	f	λ_{abs} nm	λ_{flu} nm	v_a cm^{-1}	v_f cm^{-1}	v_a-v_f cm^{-1}	φ_f
**Hexane**	1.90	1.375	0.0012	522	660	19157	15151	4006	0.83
**p-xylene**			0.003	528	682	18939	14663	4276	0.51
**triethylamine**			0.048	523	670	19121	14925	4196	0.32
**Butyl ether**	3.08	1.399	0.096	523	690	19120	14492	4628	0.31
**Isopropyl ether**	3.88	1.368	0.145	521	716	19194	13967	5227	0.09
**Ethyl ether**	4.34	1.352	0.167	517	727	19342	13755	5587	0.04
**ethyl acetate**			0.2	511	784	19569	12755	6814	0.01
**THF**	7.58	1.407	0.210	518	792	19305	12626	6679	<1%
**DCM**	8.93	1.424	0.217	515	817	19417	12239	7178	<%
**crystalline powders**				527	701	18975	14286	4689	0.487

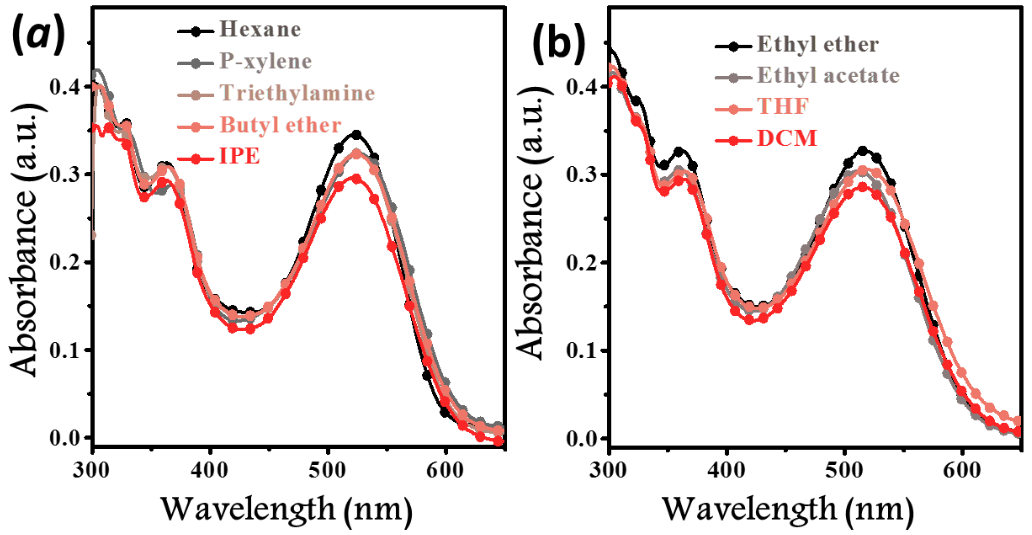

Figure S1 The UV absorption spectra of **BPMT**, measured in the different solvents with increasing polarity (the orientational polarizability of solvents, Δ*f*, -hexane: ~0; p-xylene: 0.003; triethylamine: 0.048; butyl ether: 0.096; Isopropyl ether (IPE): 0.145; Ethyl ether: 0.167; ethyl acetate: 0.200; tetrahydrofuran (THF) : 0.210; dichloromethane (DCM) : 0.218; (**Table S2**, Supporting Information)

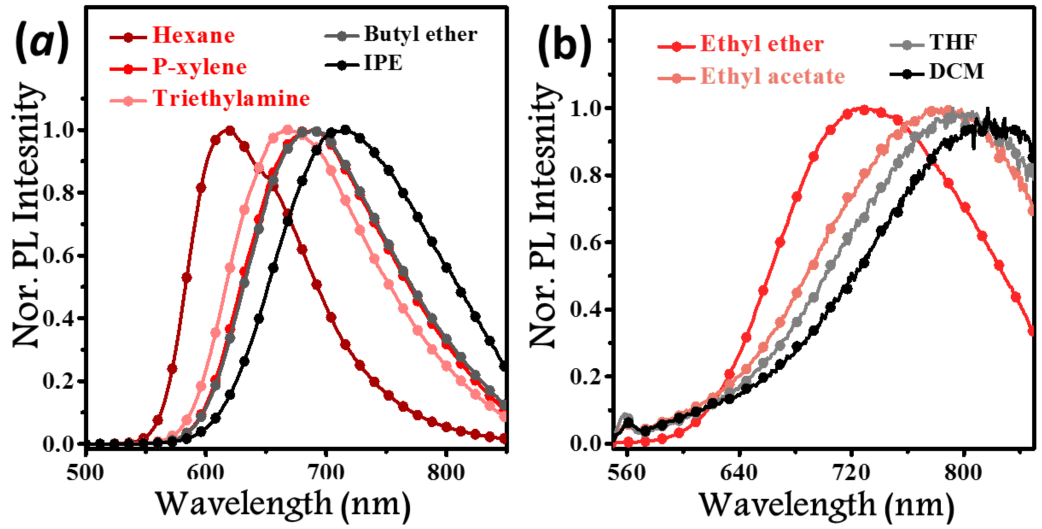

Figure S2 The PL spectra of **BPMT** in different solvents (10 μM,). The excitation wavelength is 480 nm.

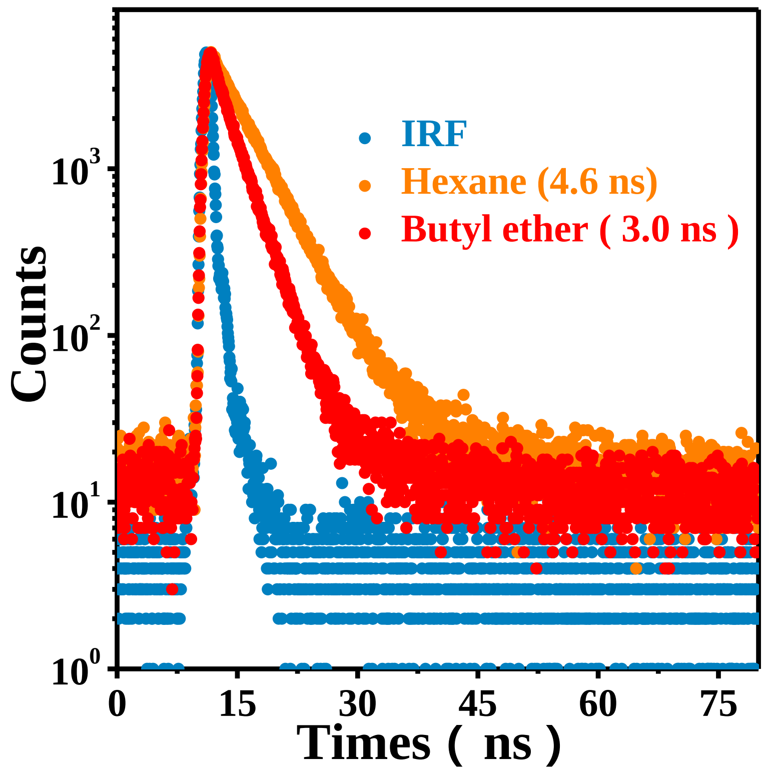

Figure S3 The fluorescence lifetime of BPMT in the Hexane and IPE (10 μM), respectively.

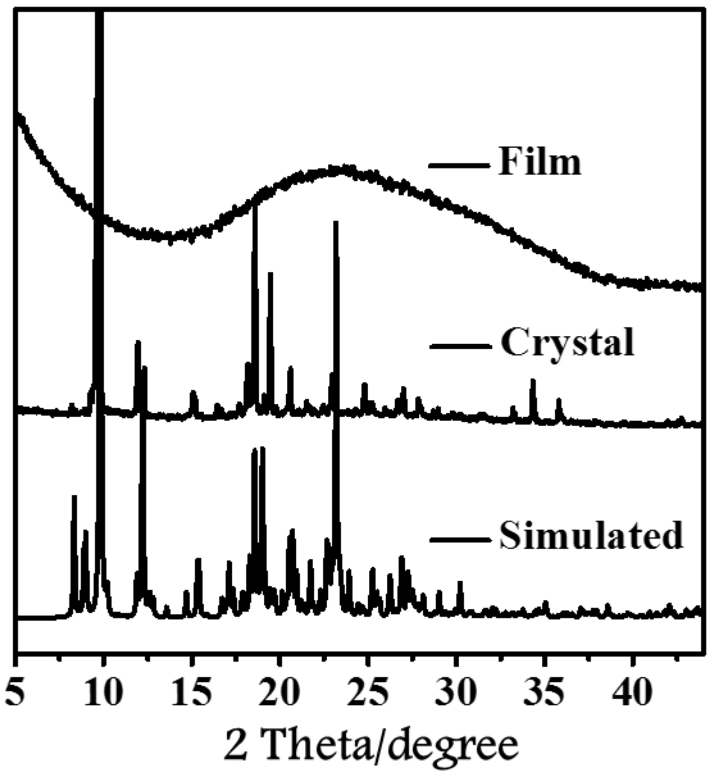

Figure S4 XRD profiles of **BPMT** in different states.

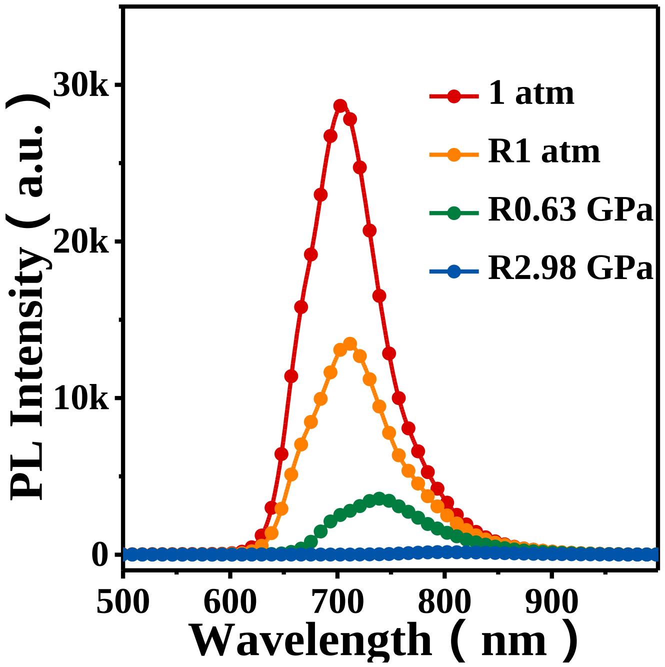

Figure S5 In-situ PL spectra of **BPMT** crystal during the depressurizing process

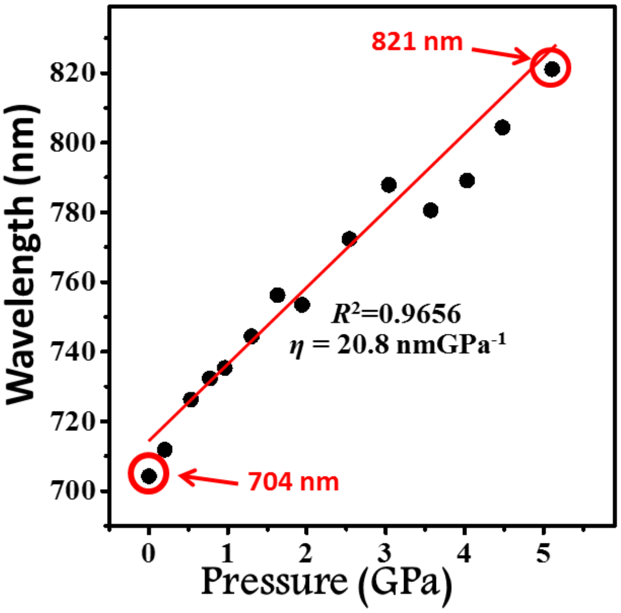

Figure S6 The corresponding plots of the relative hydrostatic pressure versus PL peaks. **Note**: The slope of the wavelength-pressure curve was calculated by the following equation [Eq. (1)]:

$$\eta=\frac{\lambda_1-\lambda_2}{P_1-P_2} \tag{1}$$

in which λ_1 and λ_2 refer to the maximum emission-peak wavelengths at pressures of P_1 and P_2, respectively.

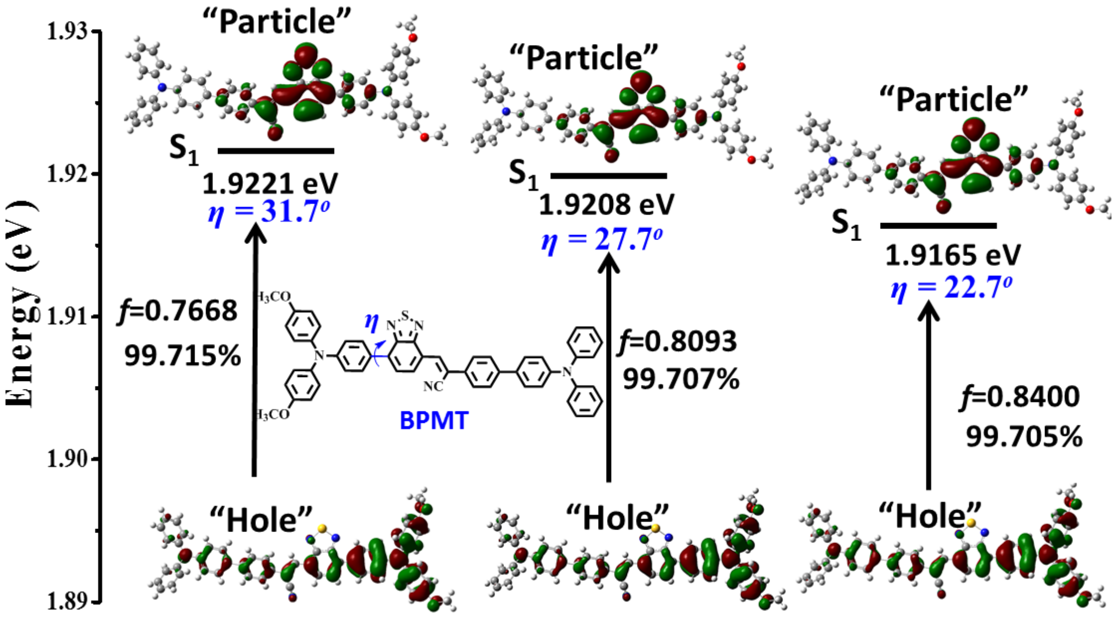

Figure S7 Excitation energies and NTO analysis of different **BPMT** molecular conformations with different dihedral angles (η), the molecular conformations resulting from single crystal structure without optimization. The percentages on the arrow are the proportions of transitions. The calculations were carried out using the TD/M06-2X/6-31g (d,p) method, and f is the oscillator strength.

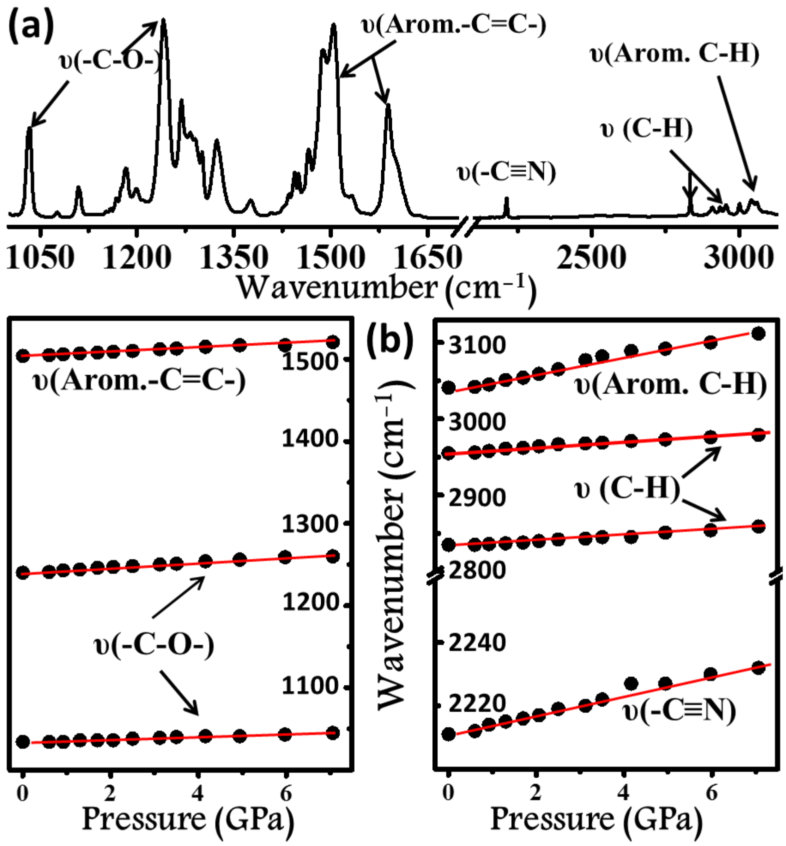

Figure S8 (a) *In-situ* IR spectroscopy of **BPMT** crystal in the range of 1000-3150 cm^{-1} at the atmospheric pressures. (b) The corresponding peak positions of -C-H, -C≡N, -C-O- and -C=C- bending and/or stretching mode as a function of hydrostatic pressure.

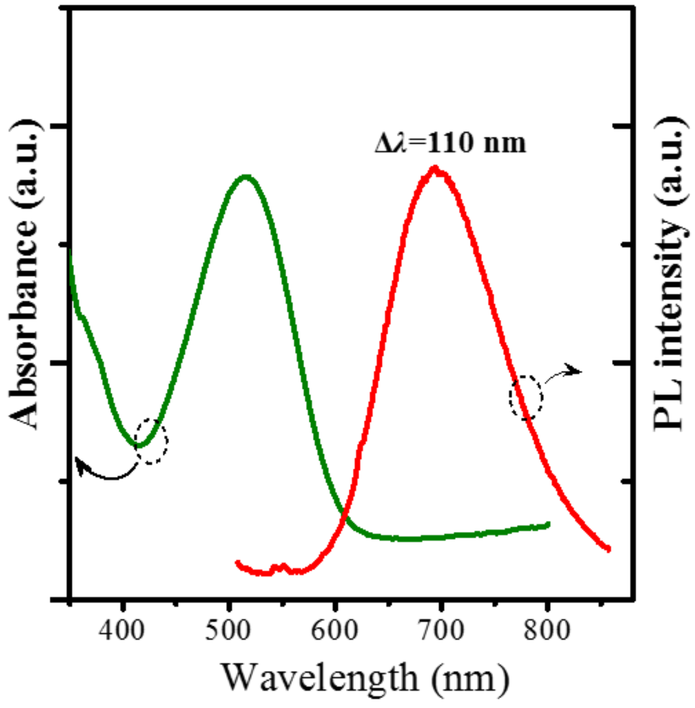

Figure S9 The absorption and PL spectra of **BPMT**-doped epoxy resin film with weight ratio of 4.8 wt%. The excitation wavelength is 480 nm.

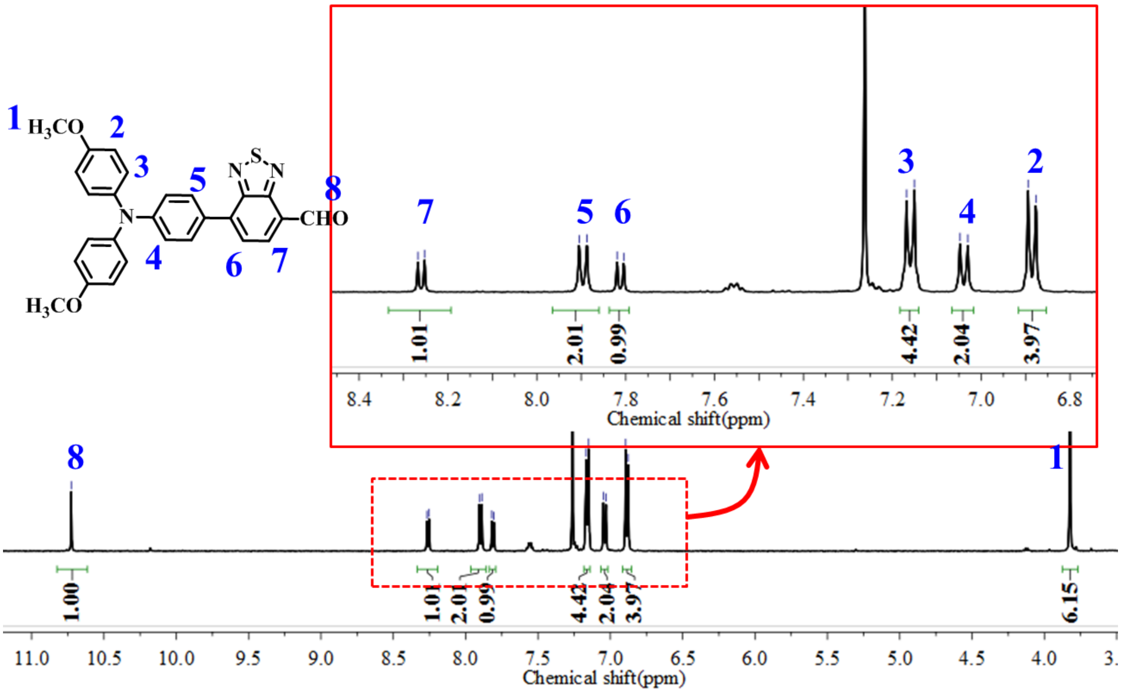

Figure S10 ^{1}**H-NMR** spectra of **MTB**.

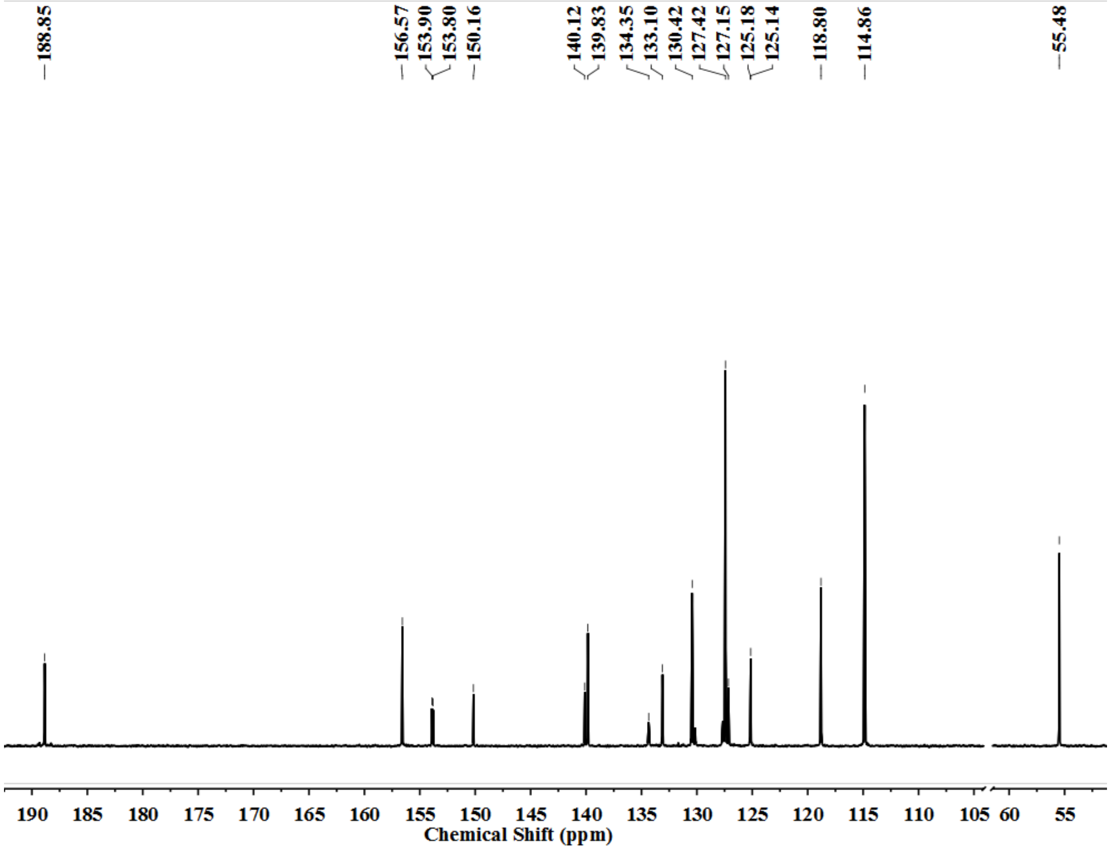

Figure S11 ^{13}**C-NMR** spectra of **MTB**.

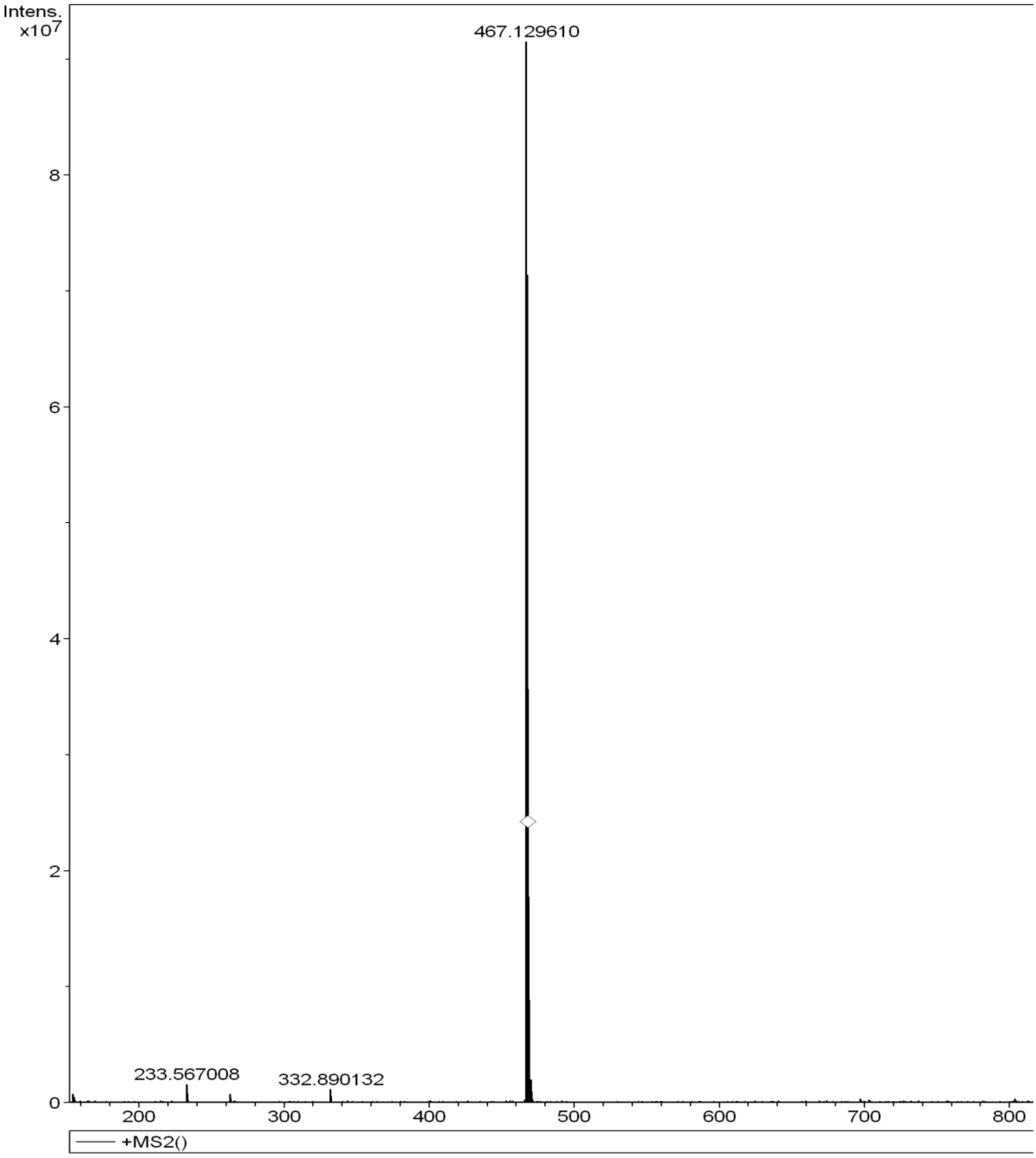

Formula	Ion Formula	Calc m/z	m/z	Diff(ppm)
$C_{27}H_{21}N_3O_3S$	$C_{27}H_{21}N_3O_3S$	467.129814	467.129610	0.43

Figure S12 MALDI-TOF-MS spectra of **MTB**.

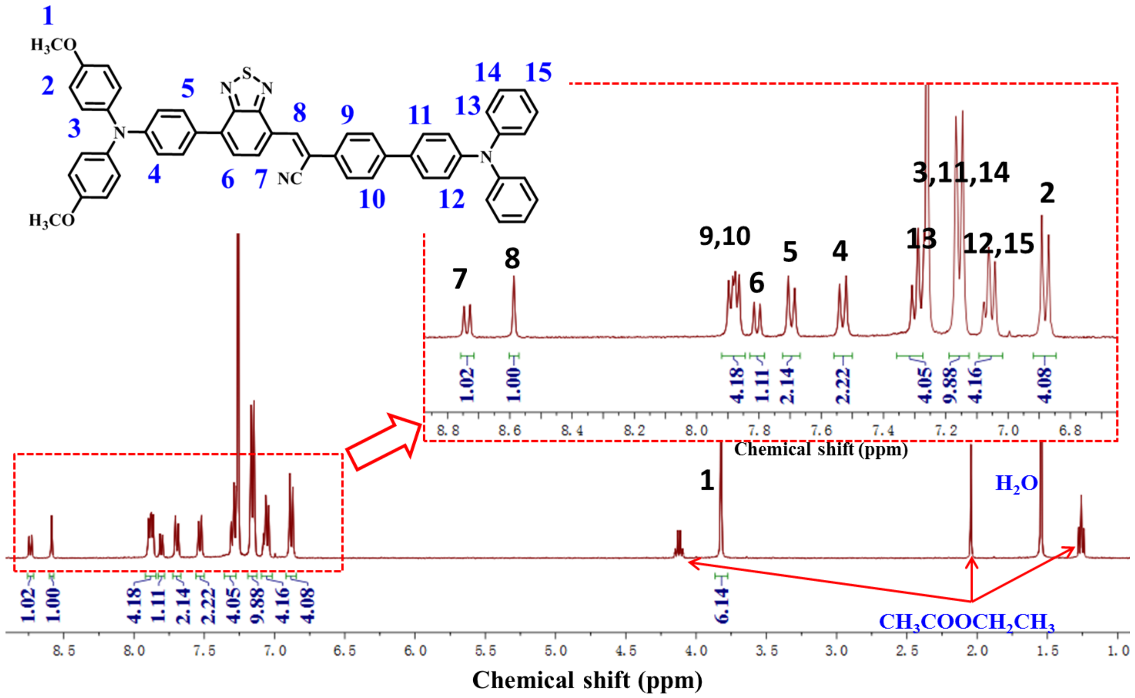

Figure S13 [1]**H-NMR** spectra of **BPMT**.

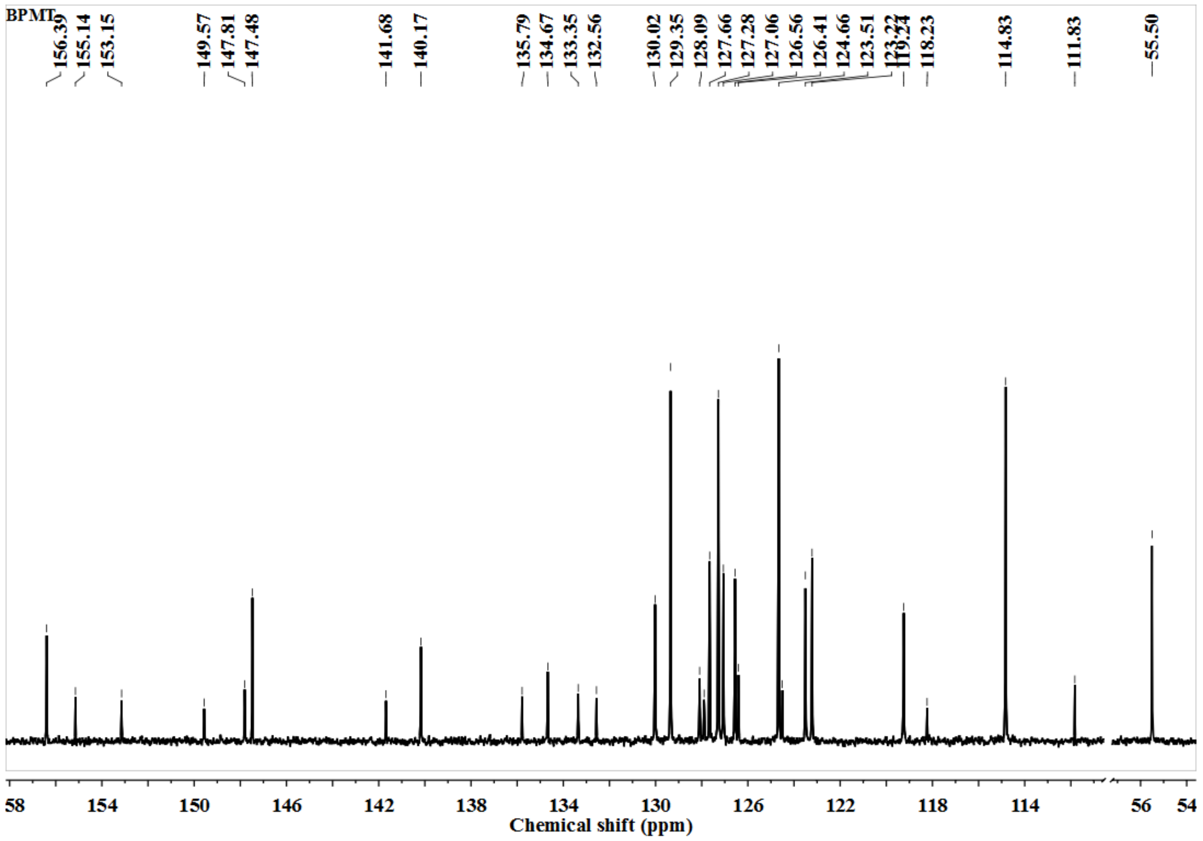

Figure S14 [13]**C-NMR** spectra of **BPMT**.

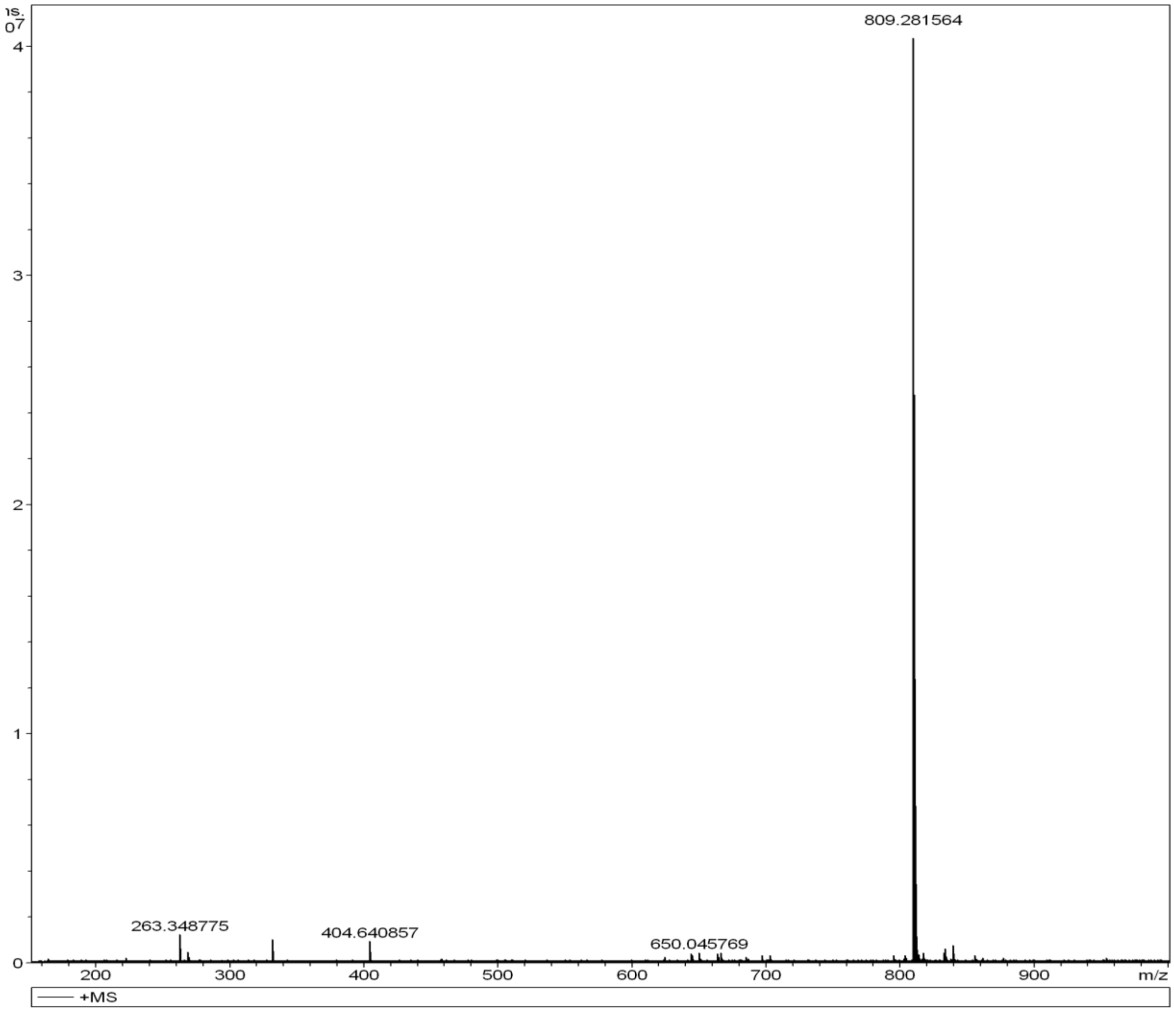

Formula	Ion Formula	Calc m/z	m/z	Diff(ppm)
$C_{53}H_{39}N_5O_2S$	$C_{53}H_{39}N_5O_2S$	809.281898	809.281564	0.37

Figure S15 MALDI-TOF-MS spectrum of **BPMT**.
